# Improvement in bilirubin influence on cholesterol efflux capacity evaluation using the immobilized liposome-bound gel beads method

**DOI:** 10.1042/BSR20230393

**Published:** 2023-06-15

**Authors:** Tsunehiro Miyakoshi, Yume Mutsuda, Yuna Horiuchi, Takahiro Kameda, Minoru Tozuka, Ryunosuke Ohkawa

**Affiliations:** 1 Department of Analytical Laboratory Chemistry, Graduate School of Medical and Dental Sciences, Tokyo Medical and Dental University (TMDU), Tokyo, Japan; 2 Department of Clinical Laboratory Technology, Faculty of Medical Sciences, Juntendo University, 6-8-1, Hinode, Urayasu, Chiba 279-0013, Japan; 3 Life Science Research Center, Nagano Children’s Hospital, Nagano, Japan

**Keywords:** bilirubin, cholesterol efflux capacity, high-density lipoprotein

## Abstract

**Introduction**: High-density lipoprotein (HDL) has a cholesterol efflux capacity (CEC) that protects against atherosclerosis. Recently, we developed an assay for CEC evaluation, named the immobilized liposome-bound gel beads (ILG) method, which is a highly accurate, simple, and safe method for CEC evaluation because it uses liposomes and BODIPY-labeled cholesterol instead of cultured cells and radioactive substances, respectively. Although the ILG method can be implemented in clinical settings, our previous study revealed that bilirubin causes a positive error in the CEC value. Therefore, in the present study, we attempted to improve the influence of bilirubin levels on the ILG method.

**Methods**: To investigate why bilirubin caused a positive error in CEC values when using the ILG method, 3D fluorescence spectra of BODIPY-labeled cholesterol and bilirubin were measured. To avoid the fluorescence emitted by bilirubin, CEC was measured using the ILG method with shifting of excitation wavelength for BODIPY-labeled cholesterol quantification. In addition, we used bilirubin oxidase to oxidize bilirubin during the incubation time of the ILG method to weaken bilirubin fluorescence.

**Results**: We found that bilirubin emitted fluorescence at the measurement setting of the ILG method. By shifting the excitation wavelength, the positive error caused by bilirubin was improved by approximately 70%. Furthermore, by utilizing bilirubin oxidase, the false-high values of CEC were improved by approximately 80%.

**Conclusions**: Bilirubin interferes with CEC assay using BODIPY-cholesterol, but we successfully improved the influence of bilirubin on CEC evaluation using the ILG method. These improvements will promote the clinical application of the ILG method.

## Introduction

High-density lipoprotein (HDL) has several atheroprotective functions such as cholesterol efflux capacity (CEC), antithrombotic activity, antioxidant and endothelial repair properties, and antiapoptotic activity [1]. To estimate the risk of cardiovascular disease (CVD), HDL-cholesterol level, which indicates the amount of HDL, has been measured at clinical sites for many years. However, it is doubtful whether HDL-cholesterol levels can truly estimate CVD risk. On the assumption that the amount of HDL is correlated with CVD risk, some studies have attempted to reduce CVD risk by increasing HDL-cholesterol levels. Despite the fact that HDL-cholesterol level was increased by cholesteryl ester transfer protein (CETP) inhibitor, CVD risk was not reduced as expected [2]. Another study showed that niacin could decrease low-density lipoprotein (LDL)-cholesterol levels and increase HDL-cholesterol levels, but they did not observe CVD risk reduction [3]. Furthermore, HDL-cholesterol levels were correlated with traditional risk factors such as age, sex, race, hypertension, diabetes, smoking, CRP, exercise, and alcohol consumption. The correlation between CVD risk and HDL-cholesterol level was canceled by adjusting for traditional risk factors [4]. These results suggested that HDL-cholesterol level could not necessarily evaluate CVD risk, and a paradigm shift from the assessment of HDL amount to its function has occurred. In this situation, CEC is one of the HDL functions that sheds light on this problem. CEC is a function in which HDL takes up cholesterol from foam cells in atherosclerotic lesions and exerts an atheroprotective effect [5]. According to a study that investigated the correlation between CEC and CVD incidence, CEC was significantly inversely associated with CVD incidence [4]. The present study also revealed that CEC was independent of traditional risk factors and HDL-cholesterol levels. From that study and other large-scale studies [6–8], CEC is recognized as an alternative biomarker for CVD of HDL-cholesterol levels. However, the conventional CEC assay methods used in these studies had some problems when applied to clinical sites. Because they need to use cultured cells [9,10], their procedures are complex, resulting in a lack of accuracy. Moreover, the use of radioactive substances in some reports [6,11,12] is also an obstacle to clinical practice. To introduce the CEC assay to a clinical site, these problems must be overcome.

We developed a novel cholesterol efflux assay using immobilized liposome-bound gel beads (ILG) [13]. The ILG method is a CEC assay suitable for clinical laboratories [14,15]. This method is a high-precision and simple assay that does not require cultured cells. As an alternative to cultured cells, the ILG assay uses liposomes immobilized on gel beads as a cholesterol supplier for HDL. The ILG method is also a safety assay because BODIPY-labeled cholesterol is used as a cholesterol tracer instead of radioactive substances, as in the conventional method. Moreover, we confirmed that the ILG method was correlated with the conventional CEC assay and can be applied to clinical sites [13].

The ILG method is a promising assay; however, an interference test showed that bilirubin caused a positive error in the CEC value [16]. The influence of bilirubin is problematic when measuring samples with hyperbilirubinemia. Even if the bilirubin level is within the normal range, the CEC value can fluctuate according to diet or exercise. These physiological fluctuations may lead to the loss of the correct CEC quantification, and bilirubin variation may affect the evaluation of CVD risk. In addition, previous significant reports on CEC using BODIPY-labeled cholesterol may also be affected by bilirubin [4,10]. If the influence of bilirubin was corrected, we could acquire a different insight into CEC. Measuring the amount of BODIPY-labeled cholesterol without the influence of bilirubin is essential, regardless of whether the ILG method or other methods are used.

In the present study, we performed BODIPY-labeled cholesterol quantification without the influence of bilirubin to improve CEC measurements using the ILG method.

## Methods

### Serum samples

Blood samples were obtained from healthy volunteers who provided written informed consent at the Tokyo Medical and Dental University. Whole blood specimens were collected directly in plastic tubes and left to stand for 15 min at room temperature (RT, 22–24°C) to allow clot formation. The sera were separated by centrifugation at 2000× ***g*** for 15 min, and the samples were stored at −80°C until use. The present study was approved by the Institutional Research Ethics Committee of the Faculty of Medicine, Tokyo Medical and Dental University (approval no. M2015-546). The present study was conducted in accordance with the World Medical Association Declaration of Helsinki.

### Preparation of interference substances-containing serum

Using a commercial kit ‘Interference Check A Plus’ (Sysmex Inc., Kobe, Japan), free bilirubin, conjugated bilirubin, hemolytic hemoglobin, or chyle was added to serum samples according to the manufacture’s instruction to prepare for interference substances-containing serum (free bilirubin: 0.0, 2.5, 5.0, and 10.0 mg/dl, and conjugated bilirubin: 0.0, 2.4, 4.8, and 9.7 mg/dl, hemolytic hemoglobin: 0, 125, 250, 500 mg/dl, chyle: 0, 422.5, 845, 1690 FTU, final concentration).

### Preparation of ILG

ILG were prepared as described previously [13]. Briefly, 10.6 mg of lecithin and 2.3 mg of cholesterol were dissolved in chloroform. Next, 15 µl of 0.5 mM 23-(dipyrrometheneboron difluoride)-24-norcholesterol (BODIPY-labeled cholesterol; Avanti Polar Lipids Inc., Alabaster, AL, U.S.A.) dissolved in chloroform was added to the solution. Chloroform was evaporated by N_2_ gas, and the steps (diethyl ether was added and evaporated) were performed twice to form liposomes that contained BODIPY-labeled cholesterol. The obtained liposomes were suspended in 14 ml of 10 mM Tris-HCl (pH 7.4) containing 150 mM NaCl and 1 mM Na_2_EDTA (Buffer A), and 0.7 g of dried sephacryl S-300 gel beads (GE-Healthcare Bio-Sciences KK, Tokyo, Japan) was added. After incubating for 30 min at RT, the cycle of freezing at −80°C and thawing in water was performed seven times to immobilize the liposomes on the gel beads. Next, the supernatant liquid that lay above the ILG was removed, and the ILG was washed with Buffer A. Finally, 10 ml of Buffer A was added and stored in the dark at 4°C.

### Preparation of apolipoprotein B-depleted serum

Apolipoprotein B-depleted serum (BDS) was prepared as previously described [17]. Briefly, whole serum and 20% polyethylene glycol (PEG) 6000 in 200 mM glycine buffer (pH 7.4) were mixed at 100:40 (v/v). After incubation for 20 min at RT, the mixture was centrifuged at 10000 rpm for 30 min at 4°C. The supernatant was collected and used as the BDS.

### CEC measurement

A cholesterol efflux assay using the ILG method was performed as previously described [13]. Briefly, 100 µl of ILG suspension and 150 µl of diluted BDS were mixed in 2-ml Eppendorf tubes. BDS was diluted with Buffer A to a final BDS concentration of 2% with the ILG suspension. The mixture was incubated in the dark for 16 h at RT. The mixture was resuspended by vortexing and centrifuged. After transferring 75 µl of the supernatant to a 96-well plate, the fluorescence intensity (FI; excitation wavelength (Ex): 485 nm, emission wavelength (Em): 538 nm, unless otherwise indicated) was measured using a Fluoroskan Ascnet (Thermo Fisher Inc., Tokyo, Japan). To normalize the variation in measurement conditions, a reference BDS was measured in each assay. The CEC value was determined as the ratio of the sample FI to the reference BDS FI. The positive error caused by bilirubin was evaluated as follows: positive error = (CEC of bilirubin additive serum)/(CEC of bilirubin nonadditive serum) × 100 − 100 (%).

### Measurement 3D fluorescence spectrums of BODIPY-labeled cholesterol and bilirubin

To ascertain whether bilirubin was misperceived as BODIPY-labeled cholesterol using the ILG method, the 3D fluorescence spectra of BODIPY-labeled cholesterol and bilirubin were measured as follows. For BODIPY-labeled cholesterol, samples were obtained by incubating BDS and ILG in the same manner as for CEC measurements. For bilirubin, BDS obtained from bilirubin additive serum was diluted with Buffer A to a final serum concentration of 3.3%, and the BDS was incubated in the dark for 16 h at RT. Fluorescence spectra of the BDS containing BODIPY-labeled cholesterol or bilirubin were measured by scanning in the range from 450 to 510 nm (Ex), and the range from 490 to 600 nm (Em) with 1 nm increments a microplate reader (Spectra Max iD3, MOLECULAR DEVICES LLC, Tokyo, Japan). The 3D fluorescence spectrum was drawn as a contour plot of excitation wavelength vs. emission wavelength vs. FI.

### Preparation of bilirubin oxidase-containing Buffer A and confirmation of bilirubin oxidation

After preparing BDS from bilirubin-containing serum (free bilirubin: 10.0 mg/dl or conjugated bilirubin: 9.7 mg/dl), each BDS was diluted with Buffer A in the presence or absence of 25 µg/l bilirubin oxidase (Asahi Kasei Corp., Tokyo, Japan). The final concentration of diluted BDSs was 3.3% as a serum. The diluted BDSs were incubated for 16 h at RT, and the absorbance spectrum from 350 to 1000 nm was measured using a spectrophotometer (UV-1280, Shimazu Corp., Kyoto, Japan). In addition, during incubation, the absorbance time course of each diluted BDS was monitored at 450 and 675 nm using a spectrophotometer every 20 min for 16 h at 25°C. To confirm whether the conversion of bilirubin to biliverdin by bilirubin oxidase could weaken fluorescence, the fluorescence spectrum of BDS incubated with bilirubin oxidase in Buffer A prepared as above was also measured. To validate that 25 µg/l of bilirubin oxidase was an adequate concentration for the ILG method, bilirubin-containing BDS was diluted with Buffer A containing various concentrations of bilirubin oxidase (2.5, 5.0, 8.0, 13.3, 25, and 40 µg/l). After incubation for 16 h at RT, the FI of each incubated BDS sample was measured (Ex: 485 nm, Em: 538 nm).

### Isolation of HDL fraction

The HDL (1.063 < d < 1.210 g/ml) fraction was isolated from healthy volunteer serum by ultracentrifugation as previously described [18].

### Confirmation of influence of bilirubin oxidase on HDL structure

To confirm that bilirubin oxidase did not affect HDL structure, we performed electrophoretic analysis of HDL incubated with bilirubin oxidase. The HDL fraction collected by ultracentrifugation was diluted with the conventional or bilirubin oxidase-containing Buffer A. The protein ratio of HDL to bilirubin oxidase was adjusted to the same ratio when BDS was diluted with bilirubin oxidase-containing Buffer A for CEC measurement by the ILG method. These samples were incubated for 16 h at RT, and electrophoresis was performed using an 8% nondenaturing polyacrylamide gel and visualized by Coomassie Brilliant Blue (CBB). To compare with the affected HDL, TritonX-100 was added to the HDL fraction to destroy the HDL particles. HDL treated with TritonX-100 was also subjected to electrophoresis, together with HDL diluted with Buffer A.

### Statistical analysis

All data are shown as mean ± SD, unless stated otherwise. Differences were evaluated using the Mann–Whitney U test or Kruskal–Wallis test with Bonferroni correction using SPSS ver. 25.0 (Chicago, Armonk, NY, U.S.A.). Statistical significance was set at *P*<0.05.

## Results

### Comparison of 3D fluorescence spectrum of BODIPY-labeled cholesterol and bilirubin

**Figure 1:**
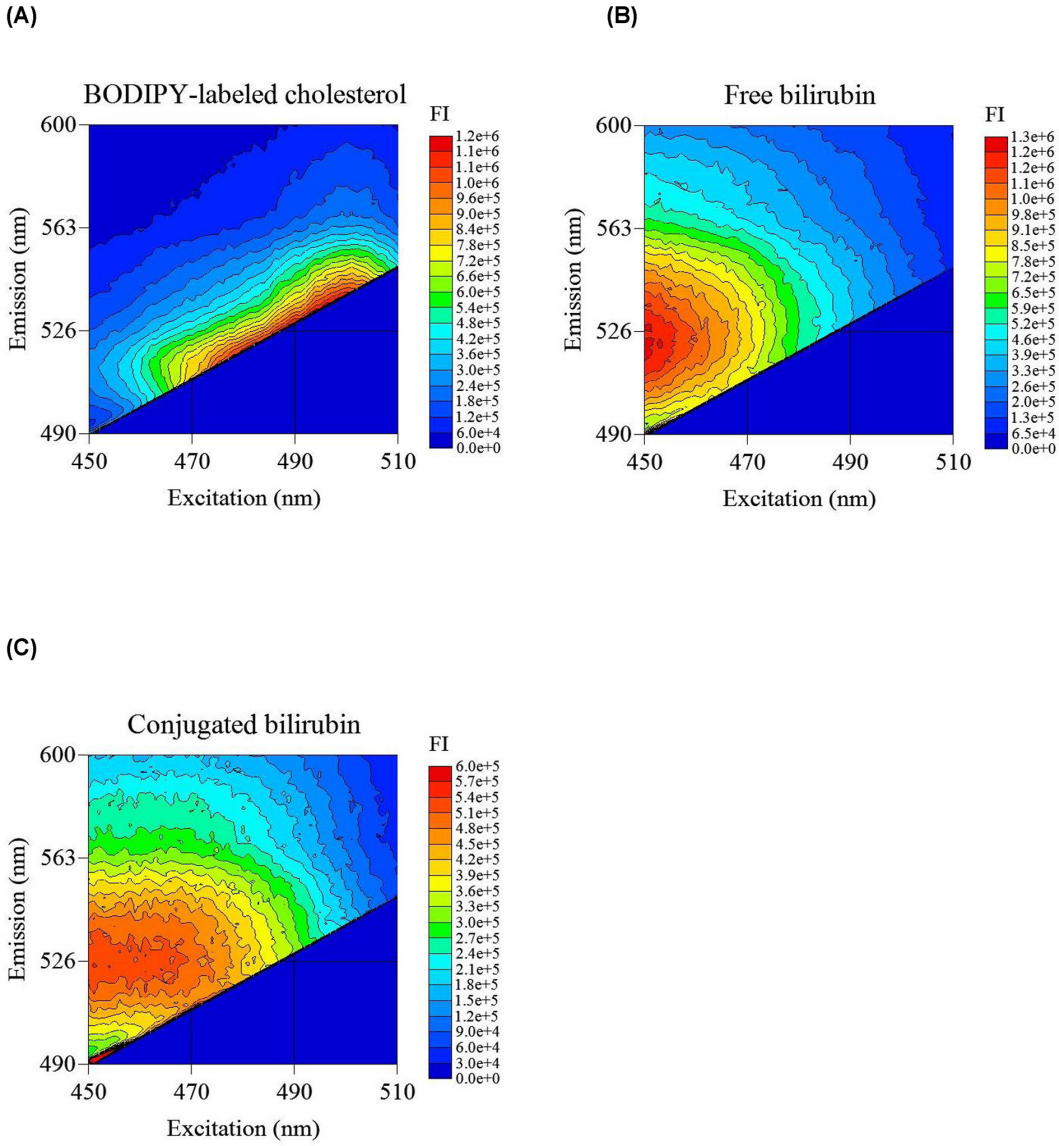
Comparison of 3D fluorescence spectrum of BODIPY-labeled cholesterol and bilirubin Fluorescence spectra of BODIPY-labeled cholesterol (**A**) in BDS obtained after the ILG assay, and of free-(**B**) and conjugated-(**C**) bilirubin in BDS after incubation were measured. Each 3D fluorescence spectrum is presented as a heatmap of FI.

To investigate the cause of the positive error by bilirubin in the ILG method, we analyzed the 3D fluorescence spectrum of bilirubin and compared its fluorescence-like property with that of BODIPY-labeled cholesterol. BODIPY-labeled cholesterol emitted fluorescence from 520 to 540 nm upon excitation from 480 to 520 nm (Figure 1A). We observed that free and conjugated bilirubin also emitted fluorescent light in widespread settings. In particular, fluorescence from 500 to 550 nm was intense from 450 to 470 nm (Figure 1B,C). From these results, at the measurement settings of the ILG method (Ex: 485 nm. Em: 538 nm), both types of bilirubin emit approximately the same intensity of fluorescence as BODIPY-labeled cholesterol. The conventional ILG method might have detected not only BODIPY-labeled cholesterol but also bilirubin.

### Improvement of positive error by bilirubin on CEC by shifting the excitation wavelength of the ILG method

**Figure 2:**
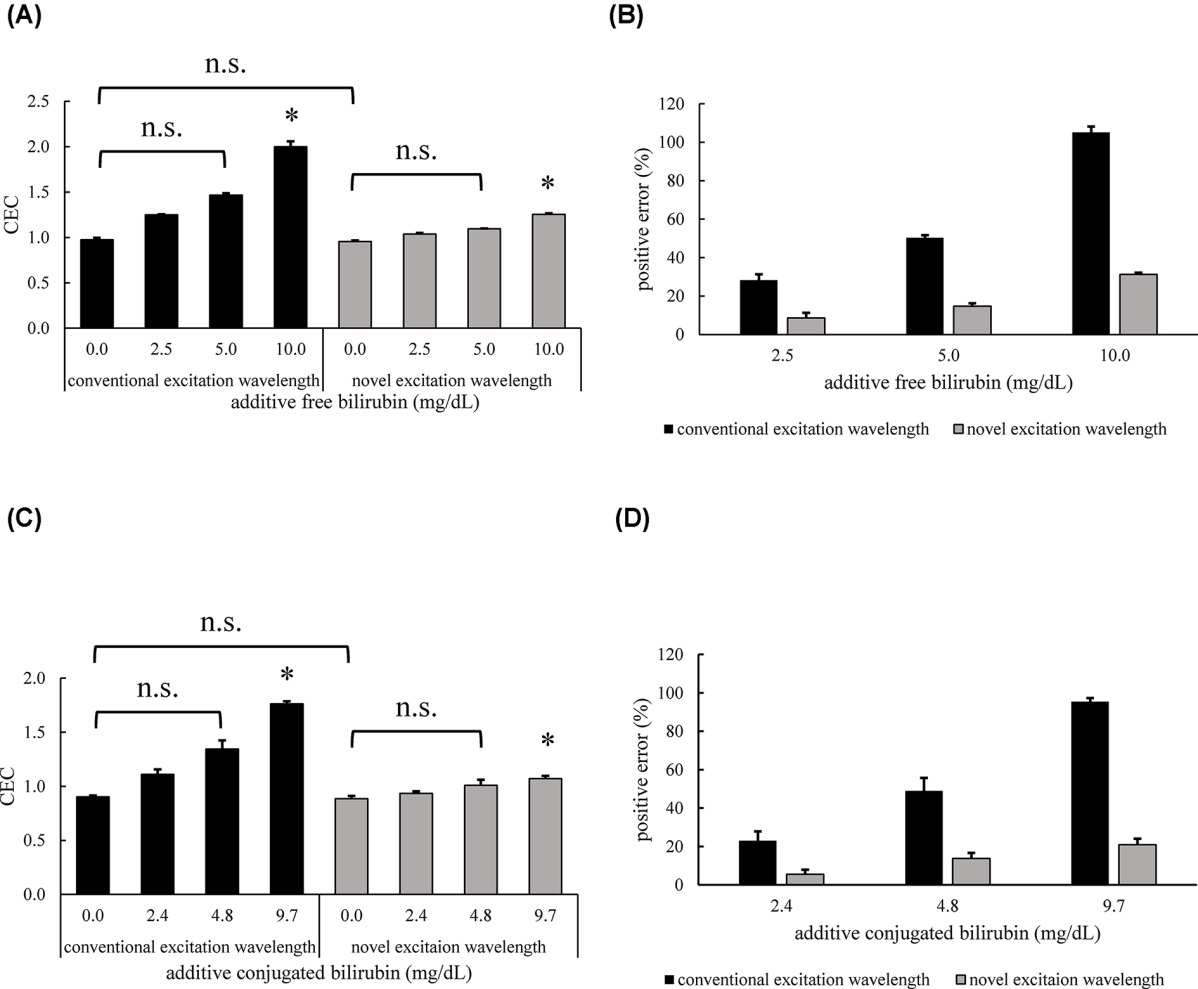
Improvement of positive error by bilirubin on CEC by shifting the excitation wavelength of the ILG method The CEC values of bilirubin additive serum and positive errors were compared using the ILG method, with the excitation wavelength set at 485 nm (conventional excitation wavelength) or 498 nm (novel excitation wavelength). CEC values at each additive-free-(**A**) or conjugated-(**C**) bilirubin concentration are presented. The positive errors for free-(**B**) or conjugated-(**D**) bilirubin, calculated by the ratio to bilirubin nonadditive serum CEC, are presented. All samples were measured in triplicate. Values are presented as the mean ± SD (*n*=3). *P<0.05 vs. control; n.s., not significant.

To avoid bilirubin fluorescence, we shifted the excitation wavelength from 485 nm (conventional excitation wavelength) to 498 nm (novel excitation wavelength) based on the 3D fluorescence spectra. First, using bilirubin nonadditive serum, we confirmed that there was no difference in CEC values between the conventional and novel excitation wavelengths (Figure 2A,C). Next, when CECs of bilirubin additive serum were measured using conventional wavelength, CEC values increased according to the additive bilirubin concentrations. In contrast, the CEC increment caused by bilirubin was gentle at a novel excitation wavelength. By shifting the excitation wavelength, the positive errors for free bilirubin and conjugated bilirubin improved by approximately 70% within 10.0 mg/dl (Figure 2B) and approximately 78% within 9.7 mg/dl (Figure 2D), respectively. However, a statistically significant difference was still observed at 10.0 mg/dl (free bilirubin, Figure 2A) or 9.7 mg/dl (conjugated bilirubin, Figure 2C). We also confirmed that the other interference substances, hemolytic hemoglobin, and chyle did not have any effects on CEC measurement with shifting of excitation wavelength (Supplementary Figure 1A,B).

### Diminishment of bilirubin by the effect of bilirubin oxidase

**Figure 3:**
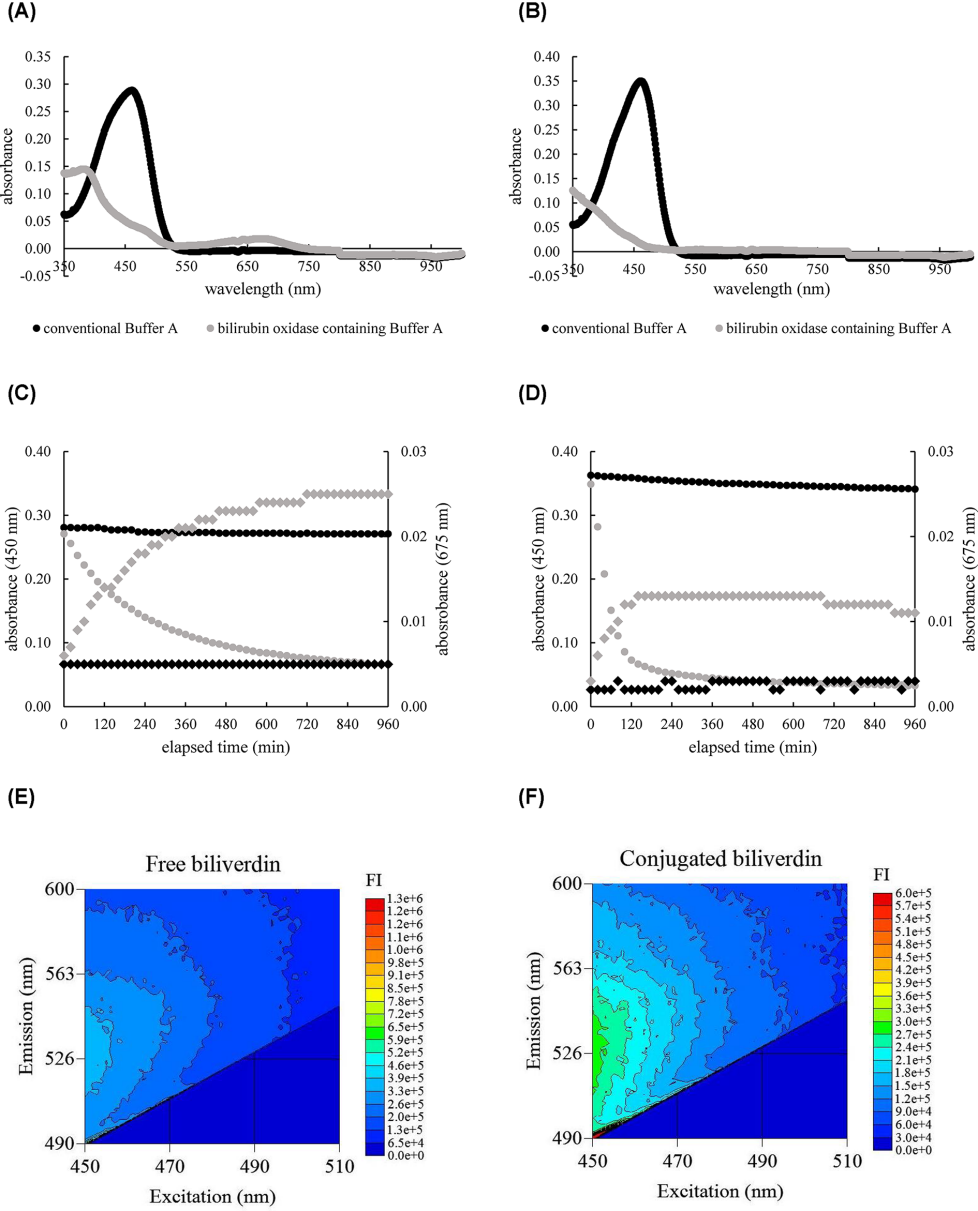
Diminishment of bilirubin by the effect of bilirubin oxidase The absorbance spectra of free-(**A**) or conjugated-(**B**) bilirubin-containing BDS incubated with conventional Buffer A and bilirubin oxidase-containing Buffer A were measured. The time courses of free-(**C**) or conjugated-(**D**) bilirubin decrement and biliverdin increment were monitored for 16 h. The black and gray markers indicate the absorbance of samples incubated with conventional Buffer A and bilirubin oxidase-containing Buffer A, respectively. Circle and diamond markers indicate the absorbance at 450 and 675 nm, respectively. The 3D fluorescence spectrum of biliverdin converted from free-(**E**) or conjugated-(**F**) bilirubin was measured. The FI at each setting is presented as a heat map. One of the results from three separate experiments is shown for each figure.

We investigated the effect of bilirubin oxidase in Buffer A bilirubin in terms of absorbance and fluorescence properties. To confirm that bilirubin oxidase converts bilirubin to biliverdin, we measured the absorbance spectrum of bilirubin-containing BDS incubated with conventional Buffer A or bilirubin oxidase-containing Buffer A. A strong peak at 450 nm, which is characteristic of the bilirubin absorbance spectrum, was observed in the spectrum of both free bilirubin- and conjugated bilirubin-containing BDS incubated with conventional Buffer A (Figure 3A,B). However, these peaks disappeared when incubated with bilirubin oxidase-containing Buffer A. The absorbance spectrum of bilirubin-containing BDS incubated with bilirubin oxidase-containing Buffer A showed absorptions from 350 to 500 nm and 550 to 750 nm, which corresponded to the biliverdin absorbance spectrum. To discriminate between the absorbance of biliverdin and bilirubin, we set the absorbance at a wavelength of 675 nm to monitor the conversion to biliverdin in the following experiment. To investigate whether this enzymatic reaction finished by the end of incubation for the efflux assay (16 h), the time courses of those reactions were monitored at 450 nm (for bilirubin, wavelength of maximum absorption) and 675 nm (for biliverdin) during the incubation. From the time course results, we confirmed that as bilirubin decreased, biliverdin increased in both free and conjugated forms (Figure 3C,D). The oxidation reaction speed for conjugated bilirubin was faster than that for free biliverdin. The 16-h incubation time was long enough because free and conjugated bilirubin were converted to biliverdin in approximately 10 and 6 h, respectively. Because the conversion of bilirubin to biliverdin was observed, we examined whether the fluorescence property of bilirubin was ameliorated by bilirubin oxidase. The 3D fluorescence spectrum of biliverdin changed dramatically compared with that of bilirubin (Figures 1B,C and 3E,F). Although biliverdin emitted slight fluorescent light, its intensity was far less than that of bilirubin. Bilirubin FI in the measurement setting of the ILG method was also weakened by the effect of bilirubin oxidase. To verify that the concentration of bilirubin oxidase in Buffer A was adequate, we investigated the correlation between bilirubin oxidase concentration and the decrease in bilirubin FI. The diminishment percentage of bilirubin FI increased proportionally to the bilirubin oxidase concentration in Buffer A (Supplementary Figure 2A,B), while it increased gently at 40 µg/l. Therefore, we determined that the optimal bilirubin oxidase concentration in Buffer A for CEC measurement was 25 µg/l.

### Influence of bilirubin oxidase on the HDL conformation

**Figure 4:**
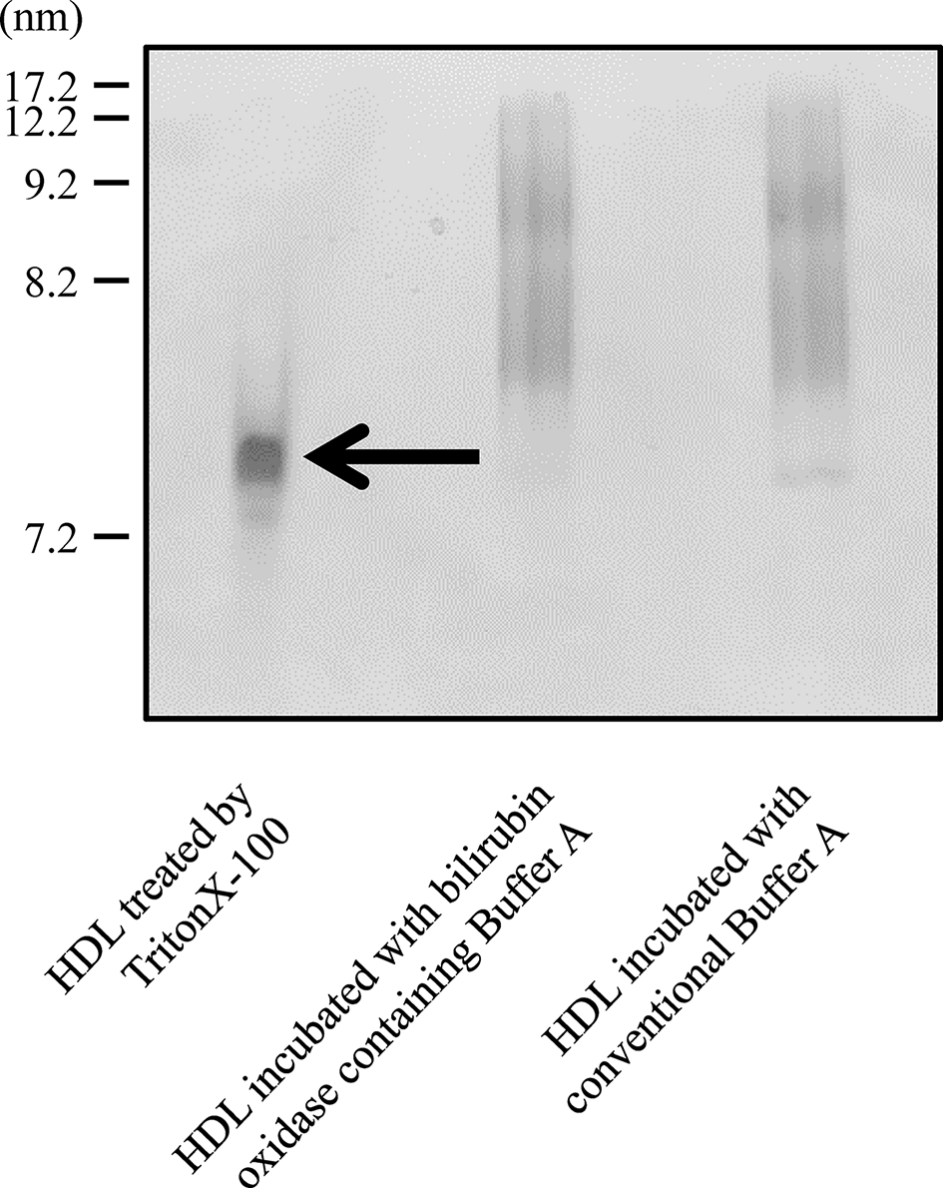
Influence of bilirubin oxidase on the HDL conformation The HDL fraction (8 µg protein/lane) incubated with conventional or bilirubin oxidase-containing Buffer A analyzed by nondenaturing polyacrylamide gel electrophoresis with CBB staining. In addition, HDL demolished with TritonX-100 was performed as the positive control. Representative data from three separate experiments are shown.

We confirmed whether bilirubin oxidase affects HDL conformation using electrophoresis on a nondenaturing polyacrylamide gel. The band patterns of HDLs incubated with conventional and bilirubin oxidase-containing Buffer A did not differ (Figure 4). However, when HDL particles were destroyed by TritonX-100, the band was found to be less than 8.2 nm in size, which was not observed in HDL incubated with bilirubin oxidase-containing Buffer A. Therefore, we could conclude that bilirubin oxidase did not have any influence on HDL structure.

### Improvement of positive error by bilirubin on CEC by utilize bilirubin oxidase on the ILG method

**Figure 5:**
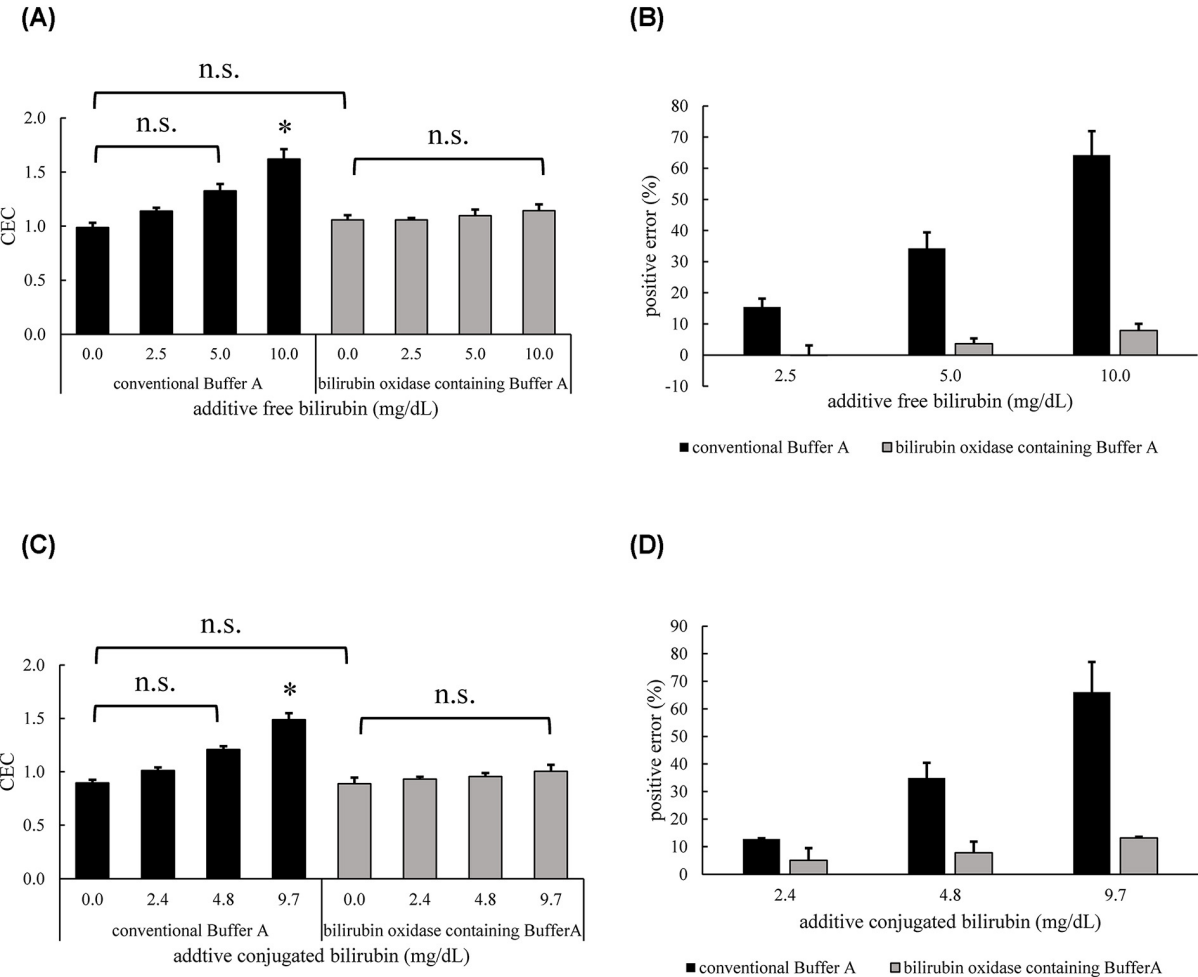
Improvement of positive error by bilirubin on CEC by utilize bilirubin oxidase on the ILG method CEC of bilirubin additive serum was measured by the ILG method with bilirubin oxidase-containing Buffer A. The CEC values at each additive free-(**A**) or conjugated-(**C**) bilirubin concentration are presented. The positive errors for free-(**B**) or conjugated-(**D**) bilirubin, calculated by the ratio to bilirubin nonadditive serum CEC, are presented. Conjugated bilirubin nonadditive sample at 1st assay was measured in duplicate. The other samples were measured in triplicate. Values are presented as the mean ± SD (*n*=3). *P<0.05 vs. control; n.s., not significant.

Since we successfully diminished the extra fluorescence from bilirubin by bilirubin oxidase, the effect of the enzyme on the actual CEC value was confirmed. Consequently, slight influences were observed when bilirubin oxidase-containing Buffer A was used for measuring bilirubin additive serum, whereas CEC values dramatically increased in a bilirubin concentration-dependent manner without the use of the enzyme (Figure 5A,C). Owing to using bilirubin oxidase, the positive errors by free- and conjugated-bilirubin were improved by approximately 88% within 10.0 mg/dl and approximately 80% within 9.7 mg/dl, respectively (Figure 5B,D). For both free- and conjugated-bilirubin, no significant differences in CEC values were observed between the four concentrations of bilirubin additive serum.

### Comparison of CEC values between conventional and improved ILG methods in healthy subjects

**Figure 6:**
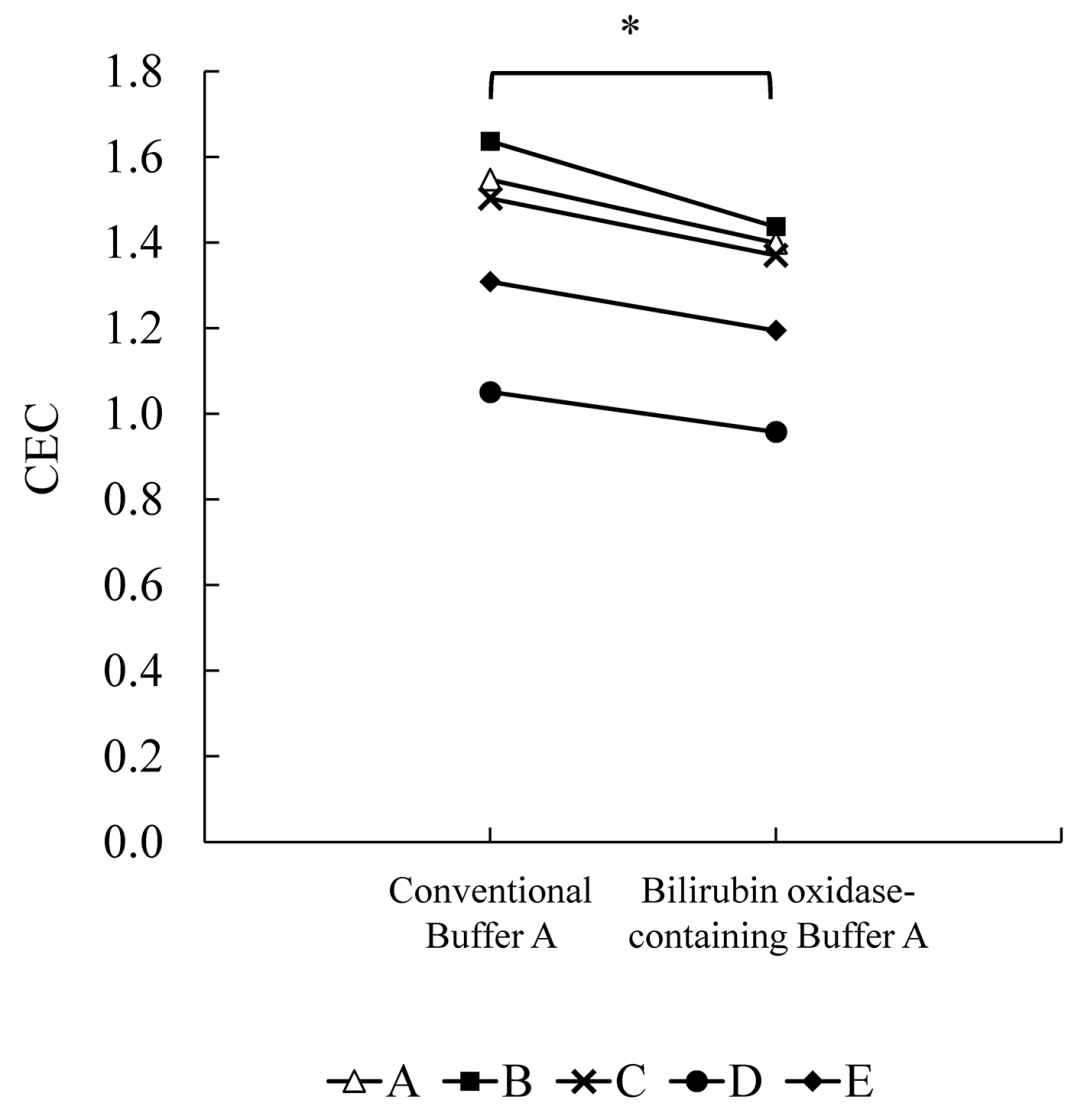
Comparison of CEC values between the conventional and improved ILG methods in healthy subjects Serum samples were collected from five healthy subjects (**A–E**), and CEC values of BDSs obtained from these sera were evaluated and compared between conventional (Buffer A only) and improved (bilirubin oxidase-containing Buffer A) ILG methods. All subjects were measured in triplicates. *P<0.05 by Wilcoxon signed-rank test

To confirm whether the improved ILG method enables a more accurate assessment of CEC value without being affected by normal range of bilirubin, we measured CEC of BDS from five healthy volunteers (subjects A–E) using conventional Buffer A and improved bilirubin oxidase-containing Buffer A. CEC values measured by the improved ILG method were decreased significantly (Figure 6). In detail, as for subject B, there was a difference of approximately 0.200 in CEC values between the conventional and improved ILG methods, which suggested that CEC value was improved by approximately 13.9%. Focusing on subjects C and D, gaps of CEC values between the conventional and improved ILG methods were 0.132 and 0.093, respectively, while the improvement ratios of subject C and D were almost same (C: 9.66%, D: 9.72%).

## Discussion

CEC is considered a suitable biomarker for CVD risk assessment. The HDL-cholesterol level evaluated at the current clinical site is supposed to indicate the amount of HDL, but CEC reflects the atheroprotective function of HDL, independent of traditional risk factors. Although our cell-free CEC method is superior to the conventional CEC assay in terms of simplicity and accuracy, the BODIPY-labeled cholesterol used in our assay was found to produce a positive error in the presence of bilirubin. We considered that the influence of bilirubin would be observed not only in the ILG method but also in other CEC assays that utilize BODIPY-labeled cholesterol. However, in previous clinical studies, BODIPY-labeled cholesterol has already been widely used to measure CEC using cell-based methods [19–22]. Therefore, we are certain that solving the bilirubin issue will contribute to related studies. Previously, we attempted to subtract the FI of the original serum bilirubin as a blank from the affected CEC by using BODIPY-free ILG. Truly, the extra bilirubin FI was canceled, but in some samples, the subtracted CEC produced a large variation [16]. In the present study, we investigated another method to improve the positive error of bilirubin in the ILG method.

The 3D fluorescence spectra of BODIPY-labeled cholesterol and bilirubin showed that bilirubin emitted fluorescent light. This light was also emitted by the excitation wavelength of the ILG method and misperceived as BODIPY-labeled cholesterol fluorescent light. We unveiled the reason why bilirubin caused a positive error in CEC measurements using the ILG method. Whether bilirubin has the ability to emit fluorescent light is unclear; however, two factors can be speculated to explain the ability of bilirubin. The first factor is the compatibility between the absorbance and excitation spectra of bilirubin. Bilirubin has a strong absorbance at 450 nm. In the present study, the 3D fluorescence spectrum showed that the excitation spectrum of bilirubin might have a peak at approximately 450 nm. This implies that bilirubin not only absorbs 450 nm light, but also causes transition and emits fluorescent light. The second factor is the chemical structure of bilirubin. Bilirubin is generated from protoporphyrin IX, which composes of heme [23]. The chemical structure of bilirubin is similar to that of protoporphyrin IX, and both substances have a conjugated structure, which is known to be involved in fluorescent light emission. Considering based on the fact that protoporphyrin IX has the ability to emit fluorescent light [24], we can conclude that bilirubin also emits fluorescence. Comparing the 3D fluorescence spectra of free and conjugated bilirubin, the peak of free bilirubin was approximately twice that of the conjugated bilirubin. We speculate that the difference in the chemical structure of bilirubin caused the difference in the fluorescence characteristics. At the measurement wavelength of the ILG method, the fluorescence intensities of free bilirubin and conjugated bilirubin were the same.

Because we revealed that the fluorescence of bilirubin caused a positive error in CEC measured by the ILG method, we shifted the measurement excitation wavelength from 485 to 498 nm. Shifting the excitation wavelength significantly improved the positive error. The FI of bilirubin was successfully reduced by shifting the excitation wavelength, whereas that of BODIPY-labeled cholesterol was maintained. Thus, we did not need to change the previous procedure and could perform this assay with only a change in the measurement setting. However, statistical significance remained with this method. Therefore, we investigated a more effective approach.

Next, we attempted to improve the positive error of bilirubin by using bilirubin oxidase. Our approach was to oxidize bilirubin using bilirubin oxidase at the step at which BDS was diluted before mixing with ILG. In general, the reagent used for bilirubin measurement contains a detergent that promotes the reaction of bilirubin oxidase. In contrast, considering the influence on HDL, we should not use detergent and diluted bilirubin oxidase with Buffer A. Thus, we investigated the reactivity of bilirubin oxidase in Buffer A. From the result of the absorbance spectrum, the bilirubin additive BDS incubated with bilirubin oxidase-containing Buffer A had absorption peaks at approximately 400 and 675 nm. This result corresponds to the absorbance spectrum of biliverdin reported previously [25], which indicated that biliverdin was generated from bilirubin by bilirubin oxidase under these conditions. With regard to the results from the 3D fluorescence spectrum, biliverdin had considerably less ability to emit fluorescent light than bilirubin. The fluorescence and absorbance characteristics of bilirubin and biliverdin were different. To utilize bilirubin oxidase in the ILG method, the bilirubin oxidation reaction should be completed within 16 h, which is similar to the incubation time for the ILG method. Hence, we monitored the decrease in absorbance at 450 nm and the increase in absorbance at 675 nm, and both free and conjugated bilirubin were eliminated during the incubation time of the ILG method. The time for conversion to biliverdin for conjugated bilirubin was shorter than that for free bilirubin, probably because of the difference in their chemical structure resulting in the difference in affinity to bilirubin oxidase. Furthermore, bilirubin oxidase-containing Buffer A did not affect the HDL structure. Collectively, we were able to establish the condition of bilirubin oxidase-containing Buffer A, which works with adequate bilirubin oxidation without destruction of HDL properties. When we used bilirubin oxidase-containing Buffer A, the positive error by bilirubin on CEC value was improved more significantly than the method with shifting measurement excitation wavelength, and the positive error by bilirubin fell within the measurement variation of the ILG method. Comparing two strategies for improving the false-positive error by bilirubin: shifting of excitation wavelength and application of bilirubin oxidase, certainly, the former method does not require any additional materials but the latter one exerts more effectively. As for pretreatment, because the oxidation reaction of bilirubin proceeds during the process that HDL pulls up cholesterol from ILG, we do not need any additional pretreatment handlings to disappear bilirubin. To summarize this discussion, we made a judge that application of bilirubin oxidase was more beneficial.

Finally, we measured CEC of BDS from healthy volunteers to confirm that the improved ILG method could be applied to actually serum measurement. Consequently, there was significant difference of CEC values by approximately 10% between with and without bilirubin influence in even healthy individuals. These observations imply that not only excessive high but also normal range of bilirubin is unignorable in the diagnosis or prediction for CVD risk. In addition, the extent of false-positive percentage depends on CEC values as well as bilirubin level. Since CEC values of patients with CVD are supposed to be lower than those of healthy subjects, the improvement rate of CEC values by the ILG method using bilirubin oxidase-containing Buffer A would be higher than those observed in the present study.

Our development in the present study enabled CEC assays using BODIPY-cholesterol to measure CEC values of hyperbilirubinemia samples, such as hepatic dysfunction or neonatal jaundice. Moreover, because we expected that especially ILG method is introduced into clinical practice as a screening test to assess the CVD risk at earlier stage, it is important to assure the applicability to various samples including icteric serum. Improvement in the positive error from bilirubin is also meaningful for patients with an even normal bilirubin range. In addition, it is not yet clear how much difference in CEC values is needed to make a diagnosis or distinguish a patient from healthy individuals. Therefore, the first important observation from the present study would make a significant contribution to solving the problem of bilirubin involvement in BODIPY-cholesterol using CEC clinical studies.

A limitation of the present study is that we measured only healthy serum and bilirubin additive serum samples to confirm the effect of the improved method. Since improvement of bilirubin influence on CEC evaluation by the ILG method using both samples was observed, further investigation is necessary to verify the improvement using patient samples and to understand how high resolution is required to evaluate the CVD risk. In the future, we will evaluate the CEC of clinical samples using the improved ILG method and verify the clinical significance of CEC measurement.

## Conclusion

In the present study, we improved the ILG method to avoid the positive errors caused by bilirubin. The ILG method has been used in clinical applications. In the future, it will be necessary to investigate the relationship between CEC values measured using the novel ILG method and the clinical condition of CVD.

## Supplementary Material

Supplementary Figures S1-S2

## Data Availability

The data used to support the findings of the present study are available from the corresponding author [Ryunosuke Ohkawa, Graduate School of Medical and Dental Sciences, Tokyo Medical and Dental University (TMDU), ohkawa.alc@tmd.ac.jp] upon request.

## Abbreviations

BDS: apolipoprotein B-depleted serum
CBB: Coomassie Brilliant Blue
CEC: cholesterol efflux capacity
CETP: cholesteryl ester transfer protein
CVD: cardiovascular disease
Em: emission wavelength
Ex: excitation wavelength
FI: fluorescence intensity
HDL: high-density lipoprotein
ILG: immobilized liposome-bound gel bead
LDL: low-density lipoprotein
PEG: polyethylene glycol
RT: room temperature
SD: standard deviation
