# Supplementary material for: Improvement in bilirubin influence on cholesterol efflux capacity evaluation using the immobilized liposome-bound gel beads method
Source: Biosci Rep. 2023 Jun 15;43(6):BSR20230393. doi: 10.1042/BSR20230393 (PMC10807951; doi:10.1042/BSR20230393)
Supplement: Supplementary Figures S1-S2 [file bsr-43-bsr20230393-supp1.pdf]

A

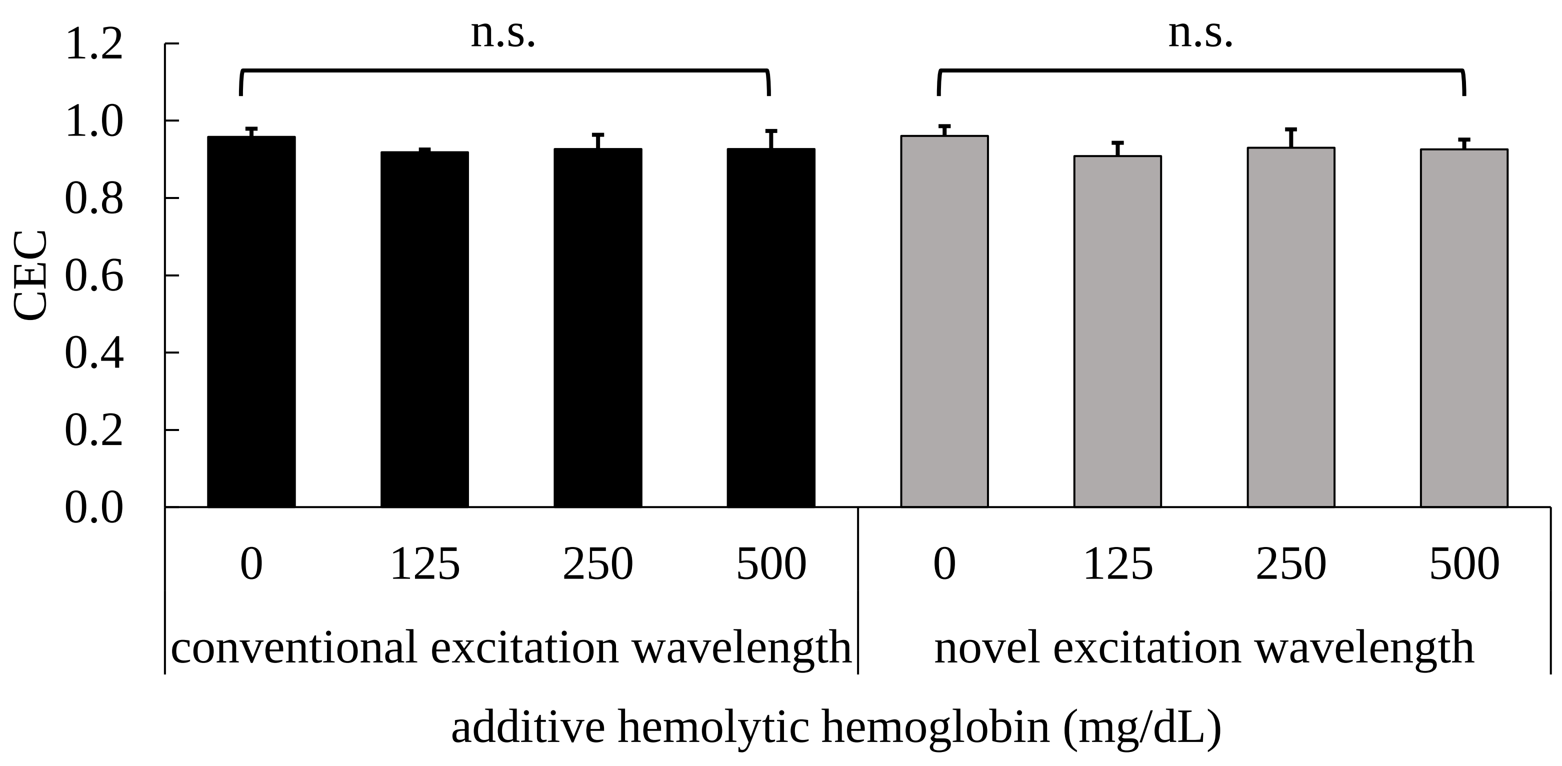

B

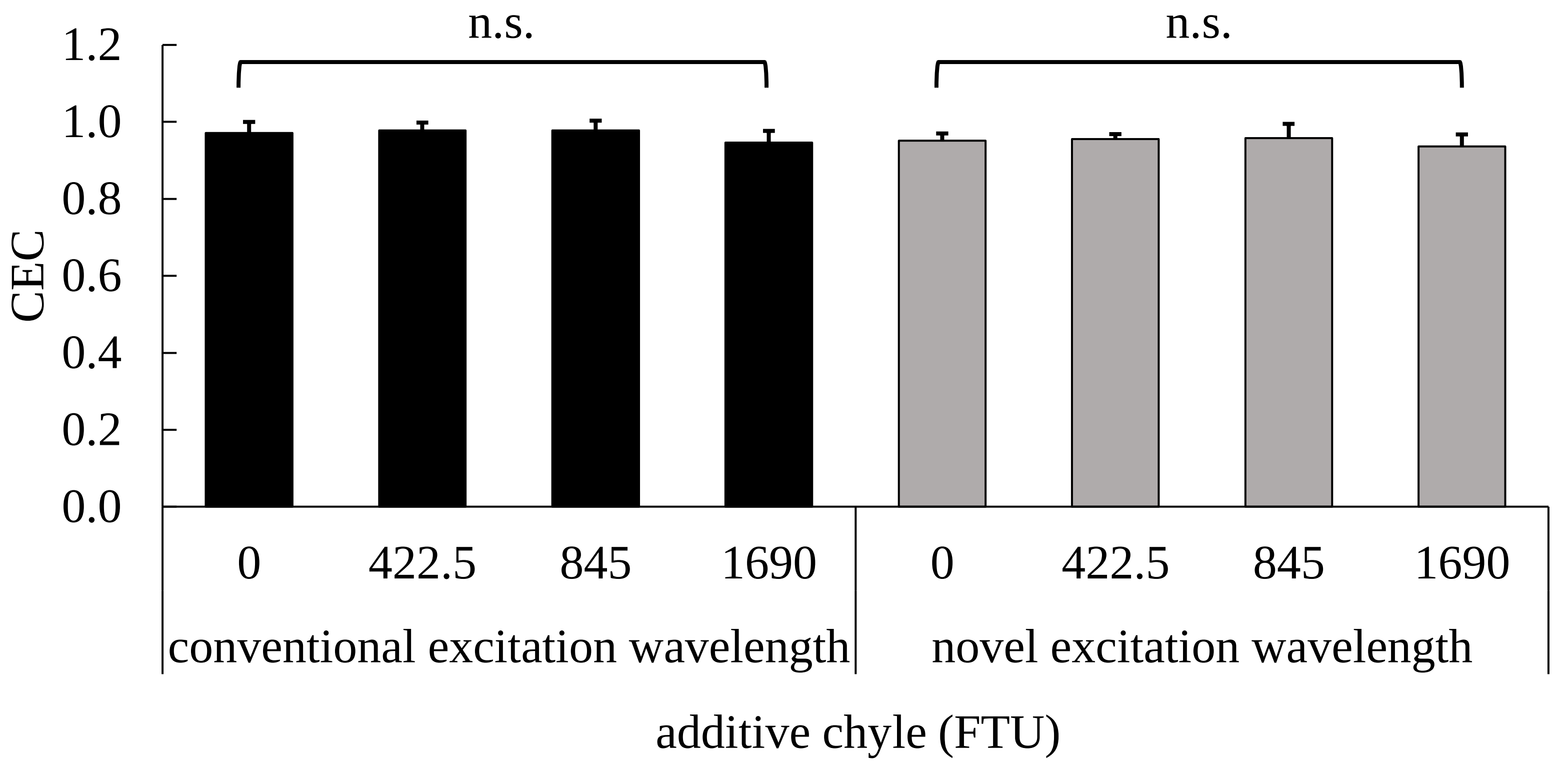

Supplementary figure 1. Influence of other interference substances on CEC measurement with shifting of excitation wavelength

The CEC values of hemolytic hemoglobin (A) or chyle (B) additive samples were compared using the ILG method, with the excitation wavelength set at 485 nm (conventional excitation wavelength) or 498 nm (novel excitation wavelength). All samples were measured in triplicates. Values are presented as mean ± SD (n = 3). n.s., not significant. FTU: Formazine Turbidity Unit

Miyakoshi et al

Supplementary 1

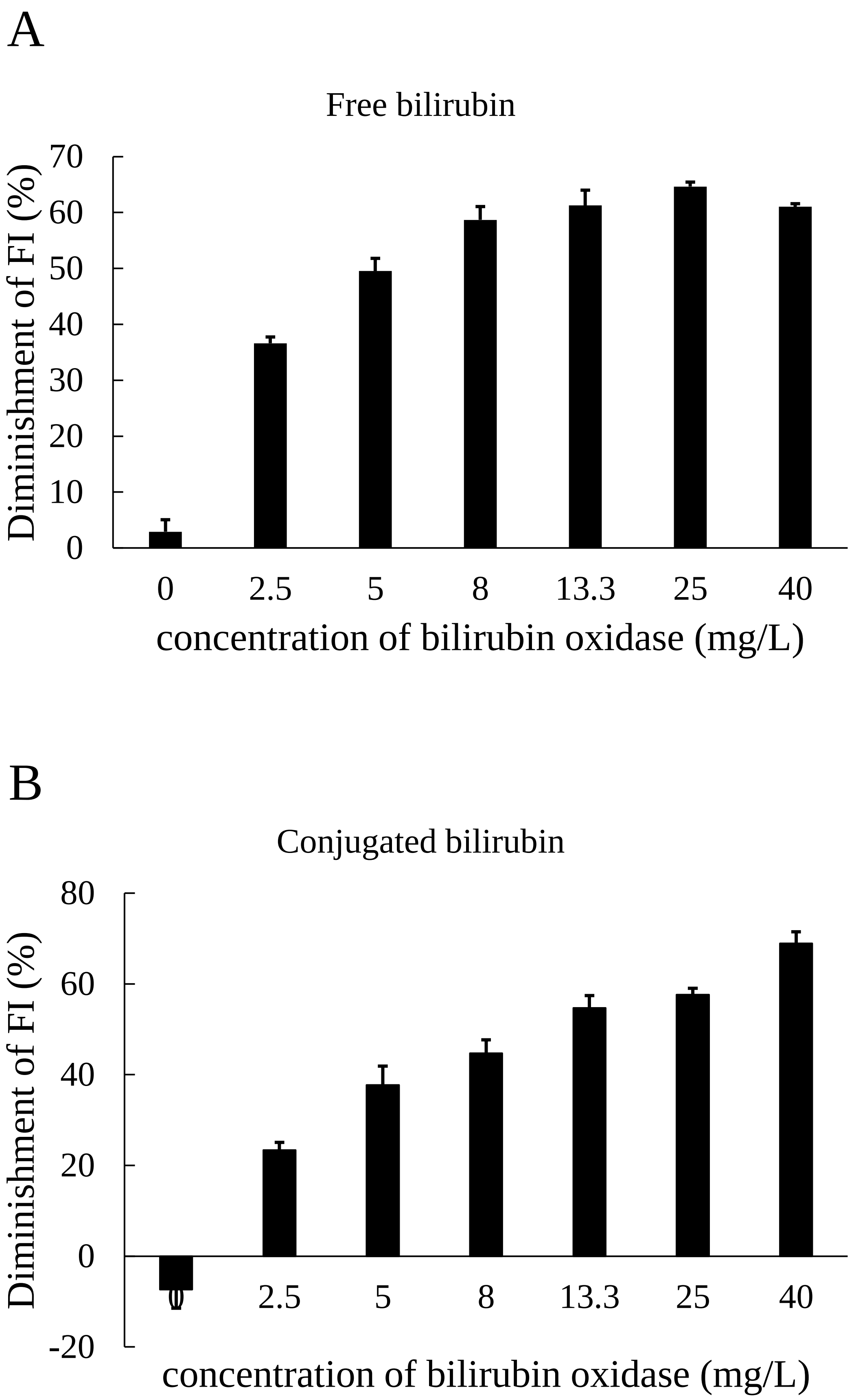

Supplementary figure 2. Diminishment of bilirubin fluorescence intensity at each concentration of bilirubin oxidase-containing Buffer A.

Bilirubin-containing BDS was diluted with Buffer A containing various concentrations of bilirubin oxidase (2.5, 5.0, 8.0, 13.3, 25, and 40 μg/L). After incubation for 16 h at room temperature (22-24°C), the fluorescence intensity of each incubated BDS sample was measured (Ex: 485 nm, Em: 538 nm). The diminishment percentage of free-(A) or conjugated-(B) bilirubin fluorescence intensity at each concentration of bilirubin oxidase-containing Buffer A is shown. All samples were measured in triplicates. Values are presented as mean ± SD (n = 3). FI: fluorescence intensity

Miyakoshi et al

Supplementary 2
